# 3D Printability of Alginate-Carboxymethyl Cellulose Hydrogel

**DOI:** 10.3390/ma11030454

**Published:** 2018-03-20

**Authors:** Ahasan Habib, Venkatachalem Sathish, Sanku Mallik, Bashir Khoda

**Affiliations:** 1 Industrial and Manufacturing Engineering Department, North Dakota State University, Fargo, ND 58102, USA; md.a.habib@ndsu.edu; 2 Pharmaceutical Sciences Department, North Dakota State University, Fargo, ND 58102, USA; s.venkatachalem@ndsu.edu (V.S.); sanku.mallik@ndsu.edu (S.M.)

**Keywords:** shape fidelity, bio-printing, hybrid hydrogel

## Abstract

Three-dimensional (3D) bio-printing is a revolutionary technology to reproduce a 3D functional living tissue scaffold in-vitro through controlled layer-by-layer deposition of biomaterials along with high precision positioning of cells. Due to its bio-compatibility, natural hydrogels are commonly considered as the scaffold material. However, the mechanical integrity of a hydrogel material, especially in 3D scaffold architecture, is an issue. In this research, a novel hybrid hydrogel, that is, sodium alginate with carboxymethyl cellulose (CMC) is developed and systematic quantitative characterization tests are conducted to validate its printability, shape fidelity and cell viability. The outcome of the rheological and mechanical test, filament collapse and fusion test demonstrate the favorable shape fidelity. Three-dimensional scaffold structures are fabricated with the pancreatic cancer cell, BxPC3 and the 86% cell viability is recorded after 23 days. This hybrid hydrogel can be a potential biomaterial in 3D bioprinting process and the outlined characterization techniques open an avenue directing reproducible printability and shape fidelity.

## 1. Introduction

Bio-printing is a revolutionary technology uses a computer-controlled 3D printing discipline to reproduce a 3D functional living tissue scaffold through controlled layer-by-layer deposition of biomaterials along with high precision positioning of cells [1,2,3,4]. A scaffold is a highly porous 3D construct that serves as a temporary structural support for growing the isolated cells, providing nutrient to new tissues, facilitating the healing process, restoring the tissue function and minimizing the wound scar [5,6,7,8,9,10]. Traditionally, a preformed solid biodegradable polymeric scaffold fabricated by casting, specifically leaching, gas forming, phase separation, melt molding, freeze drying [11,12] and electrospinning [13] is seeded with a cell suspension in tissue engineering. However, these techniques allow minimum control over pore size, scaffold geometry, interconnectivity and spatial distribution. Hence, less biomimicry is achieved [14].

To regenerate the tissue, the fabricated scaffold should support certain criteria, such as facilitating cell migration, proliferation and differentiation, removal of the waste component and finally stimulating vascularization [15,16]. Designing the scaffold architecture especially with porosity has a positive impact on cell survivability, proliferation and migration [17,18,19,20,21]. In terms of viability, the porous cell-laden scaffold shows almost 30% more viability than non-porous scaffold [22]. The porosity in scaffold facilitates the cell attachment, migration, differentiation, proliferation and extracellular matrix (ECM) production [23,24,25,26]. Therefore, the 3D scaffold architecture should be designed addressing the tissue-specific structural, mechanical and biological constraints [27,28,29].

3D bio-printing processes provide spatial control and repeatability of material deposition for attaining the design specific tissue scaffold in 3D. Three common bioprinting strategies [4] such as inkjet bioprinting [30,31], electro-hydrodynamic jetting [32,33,34,35], extrusion-based bioprinting [36,37] and laser-assisted bioprinting [38,39,40] are used to fabricate the 3D tissue scaffolds. Among them, the extrusion-based system is compatible with a diverse range of materials printing including hydrogels, biocompatible copolymers and their composition including heterogeneous bio-ink and cell spheroid [41]. Building tissue construct with cell-laden bio-ink is recently reported [42,43]. However, low extrusion pressure needs to be applied on cell-laden bio-ink to increase the cell viability [44]. A wide range of biomaterials, viscosity range from 30 mPa/s to 6 × 10^7^ mPa/s is suitable for simpler drive-control pneumatic extrusion system [45]. However, mechanical dispensing system that is, screw extruder can deliver material with higher viscosity.

Even though the actuation accuracy of extrusion based fabrication system is in micron level, the shape fidelity of the printed scaffold structure is still a challenge. Thus, identifying appropriate biomaterials to fabricate the 3D controlled porous structure using bio-AM to ensure shape fidelity and mechanical integrity is an active area of research. A number of characteristics, that is, biocompatibility, printability, degradation kinetics and byproducts should be considered before selecting biomaterials for scaffold fabrication [1]. Various types of natural hydrogel, that is, sodium alginate, gelatin, chitosan, collagen, fibrin [46] and synthetic polymers, that is, polycaprolactone (PCL), polyethylene glycol (PEG) and polylactic acid (PLA), are used as biomaterials during scaffold fabrication [47]. During and after the scaffold fabrication, it serves as a synthetic extracellular matrix (ECM) and creates cell-friendly 3D micro-environment to support the encapsulated cells [48]. Due to the containment of adjustable physical and chemical properties, hydrogels become one of the major candidates in tissue repair and drug delivery [49]. Moreover, the micro-environment having higher water content makes hydrogel especially attractive for cell delivery and encapsulation [50]. Therefore, cell encapsulation into the hydrogels is a favorable method towards overcoming the challenges of providing high cell density, achieving uniform cell distribution and ensuring suitable microenvironment for the cell.

Sodium alginate is a seaweed-extracted naturally derived polysaccharide, has been used in 3D bio-printing for several years because of its good biocompatibility. The inclusion of calcium ions directs cross-linking of the carboxylate groups of sodium alginate to achieve excellent gelation without hampering biocompatible environment [51]. Various cell types such as fibroblast [52], myoblast, endothelial [53], chondrocytes [54] and schwann [55] have been encapsulated into alginate for small scale culturing. The dissipation of Ca^2+^ ions in physiological conditions results in water-soluble, nontoxic alginate and eventually the gradual degradation of Ca-alginate gel [56]. However, achieving the intricate internal porous architecture with predefined shape, size and dimensional integrity using hydrogels material is limited. The viscosity of the hydrogel material becomes a conflicting characteristic to achieve its functionality. For example, the lower viscous hydrogel precursor, that is, less than 300 cps limits the mechanically stable structure [57]. Whereas increasing the viscosity of the hydrogel (≤100,000 cps) will bring the mechanical integrity but reduces the cell viability and proliferation [58].

To fabricate scaffold with accurate pore size and geometry, other biomaterials are often mixed with alginate to prepare a hybrid hydrogel. The hybrid hydrogel enables to achieve physical gelation characteristics and solidified filament morphology after extrusion to support the successive layers. Besides, improved cell activities and cell-friendly microenvironment that is, proliferation, migration and differentiation has been reported with hybrid hydrogel [59]. A clay-based bio-ink composed of laponite nano-clay, alginate and methylcellulose have been developed demonstrating the printability and cell viability [60]. PLA nano-fiber is mixed with alginate to fabricate knee meniscus with hASC [61]. The result shows better cell proliferation and ECM production in the hybrid filament than a pure alginate filament. In another work, methylcellulose was used with alginate to fabricate clinically relevant scaffold and the result was comparable with pure alginate scaffold [62]. Methylcellulose gives better gelation and mechanical properties to achieve clinically relevant scaffold. Gelatin has been used with alginate in several reported works. The ratio of alginate and gelatin concentration is optimized for better printability and good cell survivability [59]. The effects of various process parameters, that is, air pressure, feed rate, printing distance on the shape fidelity of the structure as well as cell survivability has been illustrated [57]. In this work, the combination of alginate and gelatin has been used as hydrogel materials. Comparison of characteristics between alginate and alginate-gelatin hydrogel in term of cell spreading, adhesion, viability has been reported where alginate-gelatin hybrid hydrogel shows comparable results [63]. A bio-ink composed of alginate and nano-cellulose named CELLINK is commercially available which presents shear-thinning and fast crosslinking features [64]. To improve the rheological properties graphene oxide (GO) is added with alginate which shows better shape fidelity through increased in hydrogen bonding [65]. A composite hydrogel including the different percentage of alginate, gelatin and hydroxyapatite has been used to demonstrate the applicability in the regeneration of bone tissue [66]. Polycaprolactone (PCL) is also combined with alginate to improve the mechanical strength of the fabricated scaffold expecting the improved functionality in bone tissue structure [67].

Carboxymethylcellulose (CMC) is a high-molecular-weight water-soluble polysaccharide used for viscosity modifier or thickener. It has been reported that the binding of CMC’s matrix protein assists in cell migration and cell attachment [68]. Moreover, alginate-CMC (Alg-CMC) hybrid hydrogel has been used to fabricate beads for various drug delivery experiments [69,70]. However, to our best knowledge, the combination of alg-CMC has not been explored for 3D bio-printing which is examined in this manuscript. The extrusion-based in-house bio-printing system is used to fabricate 3D scaffold structures with special features. Dispensing material used in this technique requires a suitable viscosity and density as well as the shape fidelity retaining capability and high cell viability during and after printing [71]. A set of systematic quantitative characterization tests such as rheological and mechanical test, filament collapse and 2D fusion test with variational pore size, the effects of air pressure and print distance on filament width are conducted to validate its printability, shape fidelity. Afterwards, 3D scaffold structures are fabricated with the pancreatic cancer cell, BxPC3 and the cell viability is recorded. The outcome of all experiments conducted and cell viability measurement imprints that this hybrid hydrogel is a favorable biomaterial for 3D bioprinting process.

## 2. Material and Method

### 2.1. Preparation of Hydrogels

**Figure 1:**
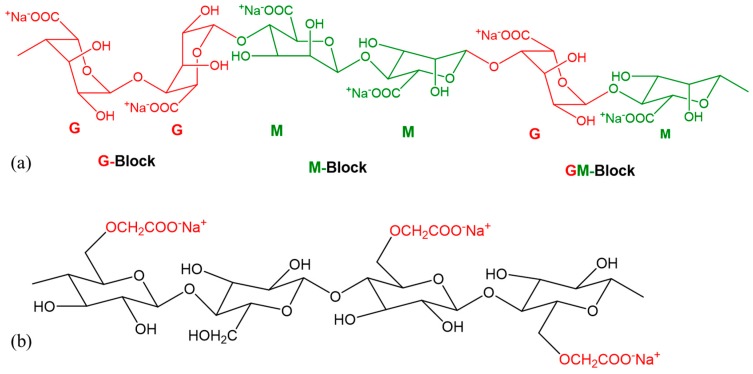
Chemical formula of (**a**) Alginate; (**b**) Carboxymethyl Cellulose (CMC).

The bio-materials used for scaffold fabrication are alginate (alginic acid sodium salt from brown algae; Sigma-Aldrich, St. Louis, MO, USA) and carboxymethyl cellulose (CMC) (Sigma-Aldrich). The chemical structures of the two materials are shown in Figure 1a,b respectively. Alginate is a common biopolymer, composed of (1-4)-linked β-Dmannuronic (M) and α-Lguluronic acids (G) as shown in Figure 1a. This material is a negatively charged linear copolymer (M and G blocks) which is soluble in the water and supports cell growth and exhibits high biocompatibility. The G-block of this material assists to form gels and GM and M blocks improve the flexibility.

**Figure 2:**
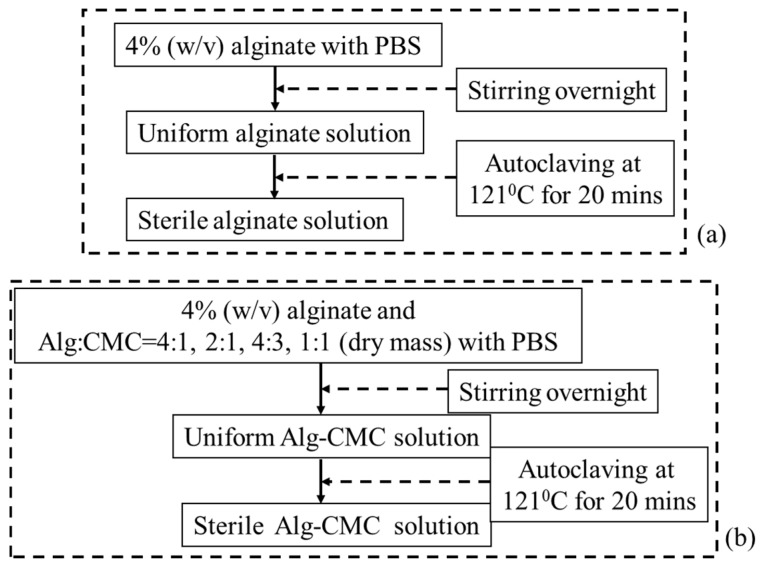
Preparation of (**a**) Alginate and (**b**) Alginate-CMC.

Carboxymethylcellulose (CMC) is an anionic water-soluble biopolymer derived naturally or through chemical reaction from cellulose. It is a copolymer of β-d-glucose and β-d-glucopyranose-2-O-(carboxymethyl)-monossdium salt which are connected via β-1,4-glucosidic bonds [72]. This material is non-toxic and non-allergenic, is widely used as thickener [73]. Each glucose monomer has three hydroxyl groups which can be substituted by a carboxyl group. More substitution of the hydroxyl group by carboxyl makes the cellulose more soluble, thicken and stable [72]. A pure alginate and mixture of alginate and carboxymethyl cellulose (Alg-CMC) solutions are prepared following the steps are shown in Figure 2.

Since both alginate and CMC are polar solute, they are soluble in water. These two materials make intermolecular action through the formation of hydrogen bonds and consequently compatible blended hybrid hydrogel. 4% (*w*/*v*) CaCl_2_ (Sigma-Aldrich) is prepared with 0.2 μm filtered deionized (DI) water and used as chemical cross-linker. The extrusion-based bio-printing system needs fast gelation. Since, alginate contains carboxyl group (–COO–), this part of the hybrid hydrogel is cross-linked with the application of divalent cation, for example, Ca^2+^. Ca^2+^ generates ionic inter-chain bridges with G and M blocks and assists to achieve fast gelation. CMC also contains carboxyl group (–COO–) which forms calcium complex with the presence of Ca^2+^. Hence, this hybrid hydrogel aids the fast formation of gel during fabrication and encapsulates the cells. Moreover, mixing CMC with alginate will increase the solution viscosity which will improve the printability.

### 2.2. Cell Culture

BxPC3, the human pancreatic cancer cells are cultured and maintained in high glucose DMEM, 2 mM Glutamine and 10% Fetal Bovine Serum (FBS) with 100 µg/mL penicillin and 100 µg/mL streptomycin (Sigma-Aldrich) in 5% CO_2_ at 37 °C incubator. The culture medium is changed twice a week. Cells at passage 5 are used for 3D bio-printing.

### 2.3. Rheological Test for Hydrogel

Rheological properties by 4% (*w*/*v*) alginate and various composition of Alg-CMC (*n* = 3) have been determined at room temperature using a Brookfield (DV-II+Pro) rotational viscometer (Middleboro, MA, USA). The shear rate, shear stress, viscosity and percentage of torque have been measured at various rotational rpm.

### 2.4. Mechanical Test for Hydrogel

The Young’s modulus of the specimens is determined using the Nano-indentation equipment. Indentation is recorded at six random spots (*n* = 6) during each run of the experiment. Measurements are carried out at room temperature with an Atomic Force Microscopy, that is, AFM (Dimension 3100, Veeco, Town of Oyster Bay, NY, USA. The indenter used in this test is a rectangular 0.01–0.025 Ωcm Anatomy (*n*) doped Si tip (RTESPA-300, Bruker, Billerica, MA, USA). The resonance frequency and an average spring constant of the indenter are about 300 kHz and 40 N/m respectively. The radius of curvature at the tip is nominally 8 nm and tip half angle is 20°. The test indentation on sapphire (Bruker, Billerica, MA, USA) determines the deflection sensitivity of the indenter. Once Young’s modulus is determined, the reduced modulus is calculated using the following equation:

(1) $$\frac{1}{E_{r}}=\frac{1-\nu^{2}}{E}+\frac{1-\nu_{i}^{2}}{E_{i}}$$

where E_r_ is reduced modulus, E_i_ = 150 GPa and $\nu_{i}=0.17$ is Poisson’s ratio of the probe respectively, E and ν are Young’s modulus and Poisson’s ratio of the sample. The Poisson’s ratio of the sample is considered as ν=0.5.

### 2.5. Scanning Electron Microscope

The microstructure of fabricated scaffold is analyzed by scanning electron microscopy (JEOL, JSM-6010LA, analytical Scanning electron microscope, Tokyo, Japan). The accelerating voltage, spot size (SS) and working distance (WD) used in this imaging are 10 kV, 50 mm and 13–14 mm respectively with magnifications of ×27, ×130, ×160, ×180, ×700 scale. The samples have been washed three times with PBS containing Ca^2+^ and Mg^2+^ and dehydrated using a gradation series of ethanol/distilled water solutions. 

### 2.6. Cell-Free Scaffold Fabrication

**Figure 3:**
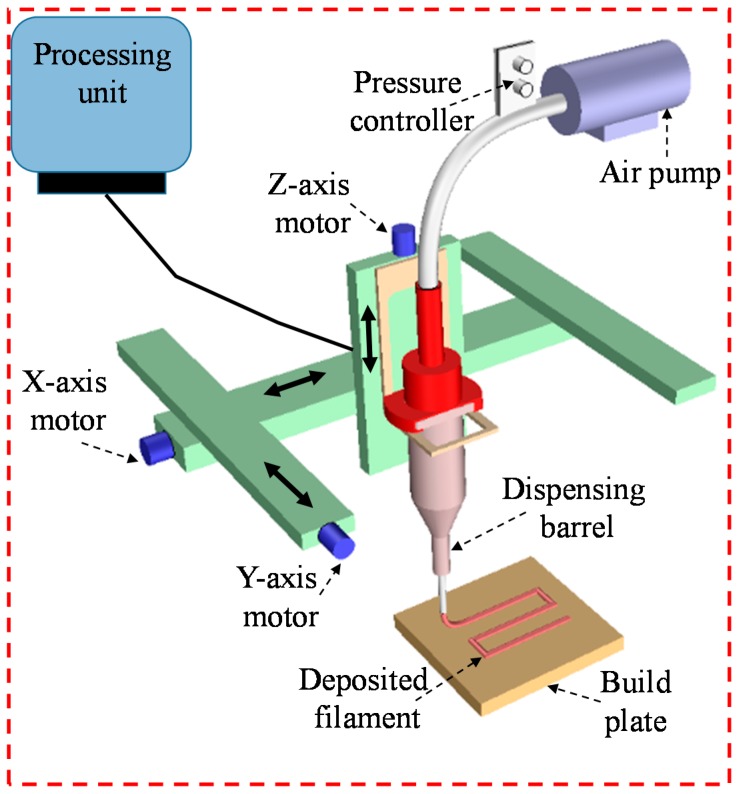
The schematic diagram of in-house built 3D bio-printer.

A three-axis in-house built 3D bio-printer is used to fabricate the acellular and cell-laden scaffold under sterile condition. The hydrogel is stored in a disposable barrel reservoir (EFD, Nordson, Westlake, OH, USA) and dispensed pneumatically through a dosing nozzle (EFD, Nordson, the inner diameter 410 µm) on a stationary print bed as shown in Figure 3. The hydrogel flow rate and width of filament are controlled by dispensing pressure, nozzle speed and print distance (i.e., the distance from the nozzle tip to build plane). The vectorized tool-path of the scaffold is programmed and converted into the machine-readable language with a visual basic based scripting language. The scaffold is fabricated layer-upon-layer where the hydrogel filaments of succeeding layer are deposited in a 0°/90° fashion into petri-dish. The spray of CaCl_2_ ensures the chemical cross-linking of the fabricated scaffold. For various compositions of Alg-CMC (4:1; 2:1, 4:3 and 1:1), 1D line, 2D grid and 3D scaffolds are printed and the filament width and pore size are measured using ImageJ software unless and otherwise stated. For each data of filament width and pore size, three random measurements (*n* = 3) are taken from random locations and the data are represented as a mean ± standard deviation.

#### 2.6.1. Filament Fusion Test

**Figure 4:**
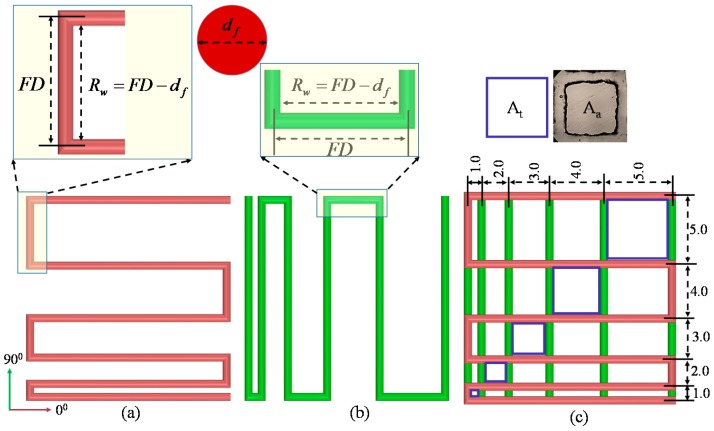
Filament deposited along (**a**) 0°; (**b**) 90° and (**c**) 0°–90°.

Four consecutive layers of various Alg-CMC compositions are fabricated layer-upon-layer without applying CaCl_2_ to conduct the filament fusion test. The fabricated scaffold follows 0°–90° pattern which captures the 2D effect and increasing filament to filament distance (FD). The range of filament to filament distance used here is 1–5 mm with 1 mm increments. After considering the filament diameter (*d_f_*), the raster width is defined as, $R_{w}=FD-d_{f}$ as shown in Figure 4. To facilitate the visualization, each composition is mixed with different colors. To avoid the undesirable surface tension, measurement is recorded from two top layers. Air pressure, nozzle speed, nozzle diameter and print distance used in this test are respectively 8 psi, 5 mm/s, 0.41 mm and 0.7 mm. Pictures of fabricated scaffolds are taken with canon (EOS Rebel T6, Melville, NY, USA) high-resolution camera right after the fabrication to avoid the unwanted material spreading. Two different factors, that is, the percentage of diffusion rate (rate of material spreading) (*Df_r_*) and printability (*P_r_*) [59] are determined respectively using Equations (2) and (3) during filament fusion test shown below. The plotted values represent the 3 repetitions of measurements for each Alg-CMC composition.

(2) $$Df_{r}=\frac{A_{t}-A_{a}}{A_{t}}\times 100\%$$

(3) $$P_{r}=\frac{L^{2}}{16A_{c}}$$

where, *A_t_* and *A_a_* are a theoretical and actual areas of pore respectively, *L* is the perimeter of the pore. The diffusion rate of a pore without any material spreading is 0 (i.e., *A_t_ = A_a_*) and for a perfect square pore, the printability is 1.0.

#### 2.6.2. Filament Collapse Test

**Figure 5:**
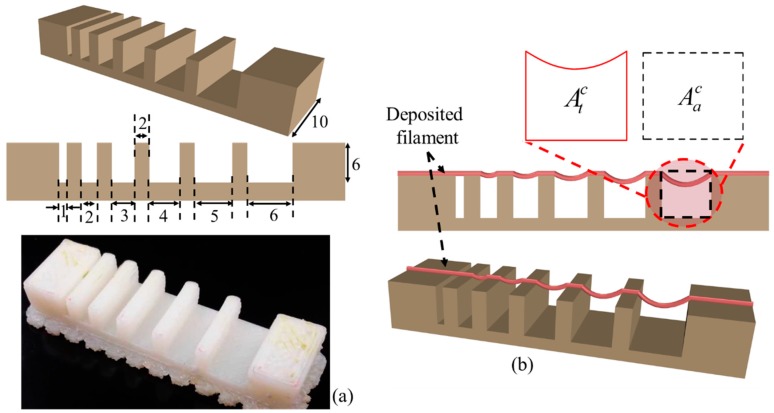
(**a**) Model and fabricated part of the platform; (**b**) Determination of collapse area factor.

The filament collapse test is conducted following our prior work [74] and the work by Therriault et al. [75]. The mid-span deflection of a suspended filament is analyzed to determine the material collapse. A platform consisting of seven pillars with a known space of 1, 2, 3, 4, 5, 6 mm are modeled with a CAD software Rhino 5.0 as shown in Figure 5a. The dimension of five pillars situated in the middle is 2 × 10 × 6 mm^3^ and the dimension of the two corner pillars is 5 × 10 × 6 mm^3^. The platform is fabricated using “Dimension 1200es” 3D printer made by Stratasys with ABS material as shown in Figure 5a. A single filament of various compositions is deposited on this platform as shown in Figure 5b.

The picture of the deposited filament is taken with canon (EOS Rebel T6) high-resolution camera right after the suspension to avoid the unwanted material deflection. Air pressure, nozzle diameter and nozzle speed used for this test are respectively 8 psi, 5 mm/s and 0.41 mm. Collapse area factor (*C_f_*), that is, the percentage of the actual area after deflecting the suspended filament with respect to the theoretical area is determined using the following equation:

(4) $$C_{f}=\frac{A_{a}^{c}}{A_{t}^{c}}\times 100\%$$

where, $A_{a}^{c}$ and $A_{t}^{c}$ are actual and theoretical area respectively as shown in Figure 5b. If the material is too viscous and unable to make a bridge between two pillars, the actual area is considered as zero and so as the collapse area factor. On the other hand, if filament does not collapse and makes a straight bridge between two consecutive pillars, then $A_{a}^{c}=A_{t}^{c}$ and consequently the collapse area factor is 100%. The plotted values represent the 3 repetitions of measurements for each Alg-CMC composition.

#### 2.6.3. Effect of Nozzle Speed, Air Pressure and Print Distance on Filament Width

For each composition of the hydrogel, a line having 10 mm length is deposited (three layers) with various nozzle speed, that is, 4, 5, 6, 7, 8, 9, 10 mm/s. The air pressure and print distance used for this test are 8 psi and 0.7 mm respectively. To determine the effect of air pressure and print distance on filament width, a line having 10 mm length is deposited (three layers) with the material composition of 4% Alg-2% CMC and 4% Alg-4% CMC. Various air pressure, for example, 5, 6, 8, 10, 12 and 15 psi is used during inspecting the effect of air pressure. The nozzle speed, print distance are used for this test is 5 mm/s and 0.7 mm respectively. Various print distances, for example, 0.4, 0.7, 0.9, 1.1, 1.3 and 1.5 mm are used to demonstrate the effect of print distance on filament width. The nozzle speed and air pressure used for this test are 5 mm/s and 8 psi respectively. For all of these three tests, the nozzle diameter is used as 0.41 mm. The width of the filament is recorded with Zeiss bright field inverted microscope for each of this test. The plotted values represent the 3 repetitions of measurements.

#### 2.6.4. Qualitative and Quantitative Test for Lateral Pore

**Figure 6:**
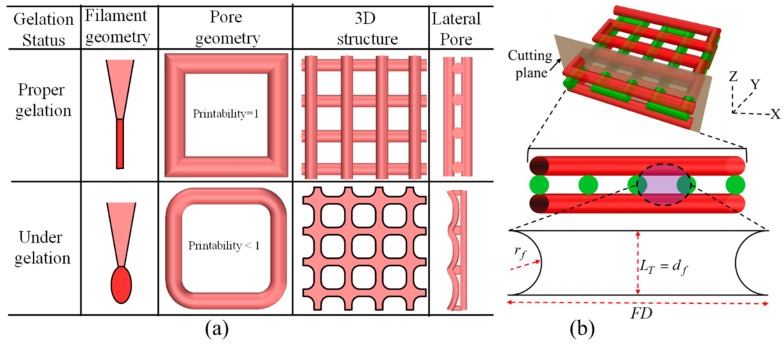
(**a**) Qualitative; and (**b**) Quantitative test of lateral pore.

Two different qualitative tests are conducted during fabrication of scaffold. Firstly, each of the compositions is suspended in open air with an 8-psi air pressure and 10 mm print distance and observed if it makes continuous filament or just droplets. If any composition makes continuous filament, this is defined as proper gelation as shown in Figure 6a. The composition making proper gelation potentially may maintain the shape fidelity of the fabricated scaffold. On the other hand, the composition making droplet during suspension is defined as under-gelation as shown in Figure 6a which loses the shape fidelity during the progress of scaffold fabrication. Secondly, the lateral porosity of the fabricated scaffold with each of the compositions is visually observed to see the difference. Scaffold fabricating with the composition having a lower viscosity will collapse in the overhang region between two consecutive filaments of the lower layer and eventually, the lateral pore may diminish.

The shape fidelity can be described quantitatively in term of the lateral porosity as shown in Figure 6b. The lateral collapse area factor, that is, the percentage of the actual lateral area of each lateral pore with respect to the theoretical lateral pore area, is determined using the following equation:

(5) $$C_{f}^{l}=\frac{A_{a}^{l}}{FD\times LT-\pi r_{f}^{2}}\times 100\%$$

where, $A_{a}^{l}$ is the actual area of the lateral pore, *LT* and $r_{f}$ are theoretical layer thickness and theoretical radius of filament respectively as shown in Figure 6b. If the lateral pore is diminished after fabrication of the scaffold, that is, $A_{a}^{l}=0$ and so as the lateral collapse area factor. The higher value of $C_{f}^{l}\to 1$ reflecting the better preservation of lateral pore and so as the scaffold shape fidelity.

#### 2.6.5. Cell-Laden Scaffold Fabrication

**Figure 7:**
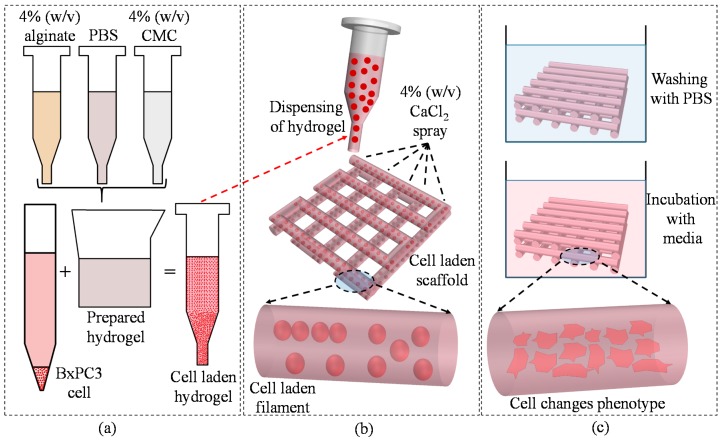
(**a**) Preparation of cell-laden hydrogel; (**b**) bio-printing of the cell-laden hydrogel; and (**c**) incubation of fabricated scaffold. Rheology.

For cell-laden scaffold fabrication, 2 × 10^6^ cells/mL are mixed with alginate and Alg-CMC solutions respectively into disposable barrel reservoir (EFD, Nordson) and dispensed pneumatically through a dosing nozzle (EFD, Nordson, the inner diameter 250 μm) as shown in Figure 7a. A 0°–90° deposition direction is followed to dispense ten layers of cell-laden scaffold with a dimension of 10 mm × 10 mm × 2.0 mm with1.0 mm filament to filament distance.

**Figure 8:**
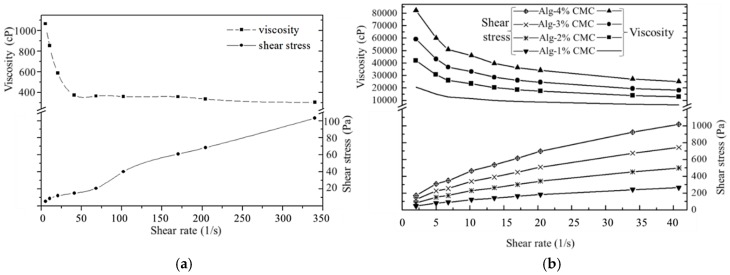
Rheological properties of (**a**) Alginate and (**b**) Alginate-CMC.

The cell-laden scaffold is preserved in 5% CO_2_ and 37 °C incubator with the same medium used for cell culture referred in Section 2.2. The medium was changed twice in a week. Bio-printing of cell-laden scaffold and incubation are schematically shown in Figure 8a,b respectively.

### 2.7. Swelling Test of Filament

The swelling test is done following the protocol described in [76]. Briefly, sample filaments with various material compositions that is, 4% alginate, 4% alginate-1% CMC, 4% alginate-2% CMC, 4% alginate-3% CMC, 4% alginate-4% CMC has length of 1 cm are printed with a diameter of 250 μm dispensing nozzle. The sample number for each material combination is *n* = 3. Before storing them in liquid media, the weight of all the filaments is recorded and denoted as dry weight (W_d_). All of the printed filaments are immersed in the culture media and incubator having ideal culture condition, that is, 37 °C and 5% CO_2_. The weights of the filaments are recorded every day up to 11 days and denoted this weight as wet weight (W_w_). Using the following equation, % of swelling rate of these filaments is determined:

(6) $$\%\;\mathrm{of}\;\mathrm{swelling}\;\mathrm{rate}=\frac{W_{w}-W_{d}}{W_{d}}\times 100$$

### 2.8. Live/Dead Assay for Cell Viability

During deposition the cell-laden filament through dispensing nozzle, the cell experiences shear stress, which could be potentially harmful to the cell. Thereby, the cell viability and cytotoxicity is conducted using LIVE/DEAD assay after the printing and at the different time period. ReadyProbes™ Cell Viability Imaging Kit, Blue/Green (Thermofisher, Waltham, MA, USA) was used following the manufacturers protocol. The filament with the cells was imaged using Lionheart FX automated live cell imager (Biotek, Winooski, VT, USA). The z-stack images are captured using 50 μm layer thickness. The protocol is defined accordingly and beacon (*n* = 5) is selected randomly. Laser power and other detector parameters are kept constant throughout the imaging of the different beacons. The percentage of viability is determined using the following equation:

(7) $$\%\;\mathrm{Viability}=\frac{\mathrm{live}\;\mathrm{cell}}{\mathrm{live}+\mathrm{dead}\;\mathrm{cell}}\times 100$$

### 2.9. Statistics

All the data are presented as a mean ± standard deviation. To evaluate the statistical significance of differences in viscosity, filament fusion, collapse tests, Young’s and reduced modulus, the distribution of values are considered to be normal. At a significance level of *p* = 0.05, a two-way ANOVA is performed to determine the statistically significant differences. Statistical software Minitab 18.0 and origin pro 5.0 are used to do the quantitative and graphical analysis.

## 3. Result and Discussion

### 3.1. Rheological Properties

**Figure 9:**
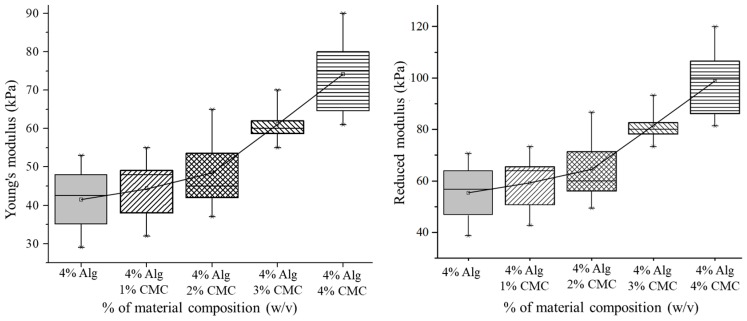
Young’s and Reduced Modulus.

The shear thinning behavior, that is, decreasing the viscosity of the fluid with an increasing shear strain of pure alginate and Alg-CMC hydrogels is revealed from the stress curves as shown in Figure 9. In case of alginate/CMC, the shear thinning effect is pronounced more which reflects the greater possibility of this hybrid hydrogel to bio-print the clinically relevant scaffold.

### 3.2. Mechanical Test for Hydrogel

**Figure 10:**
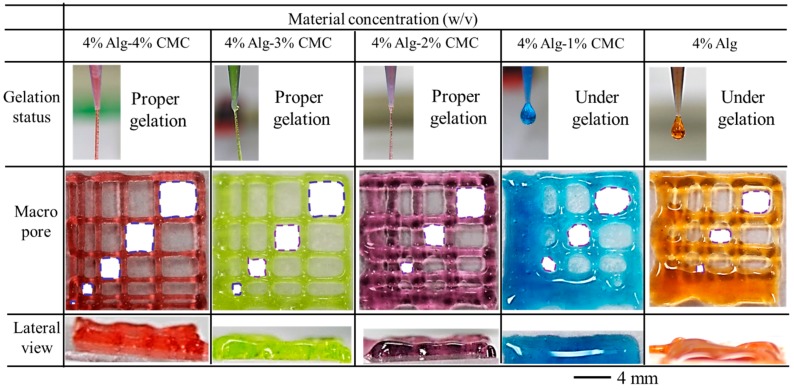
Filament fusion test.

Mixing of CMC with alginate has increased the Young’s and Reduced Modulus of the blended hydrogel as shown in Figure 10. The mean value of the response, that is, Young’s modulus of various material concentrations is significantly different (*p*-value = 0 with 95% confidence interval). Incorporation of CMC into alginate increases the crosslinking density by increasing the Ca^2+^/–COO^−^ ionic interaction as shown in Figure 2. Moreover, the hydrogen bond between the same –COO^−^ ion of alginate and CMC makes the hybrid hydrogel stronger. Hence, with the increment of CMC percentage leads the upward trend of the Young’s and reduced modulus as shown in Figure 9.

### 3.3. Filament Fusion Test

**Figure 11:**
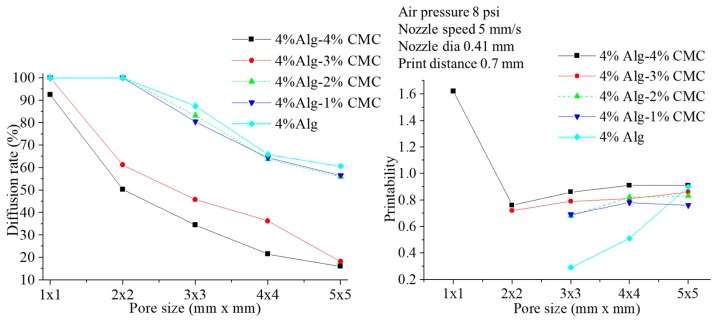
Diffusion rate and printability in filament fusion test.

To evaluate the effect of filament fusion and pore closure for each composition of the hydrogel, the area of the designed variational pore of the fabricated scaffold is measured as shown in Figure 10. The diffusion rate and printability of each pore are determined following the Equations (2) and (3) respectively. The diffusion rate shows decreasing trend where the printability shows an increasing trend with increasing the pore size for each composition of material as shown in Figure 11. Qualitatively, the 4% Alg-4%CMC shows good pore size and geometry than other material compositions. The quantitative analysis of diffusion rate validates that the composition of 4% Alg-4% CMC shows minimum material spreading, that is, minimum diffusion rate than other compositions. The range of printability of 4% Alg-4% CMC is 0.78 to 0.92 for the pore size 2–5 mm which demonstrates almost square geometry of the pore.

### 3.4. Filament Collapse Test

**Figure 12:**
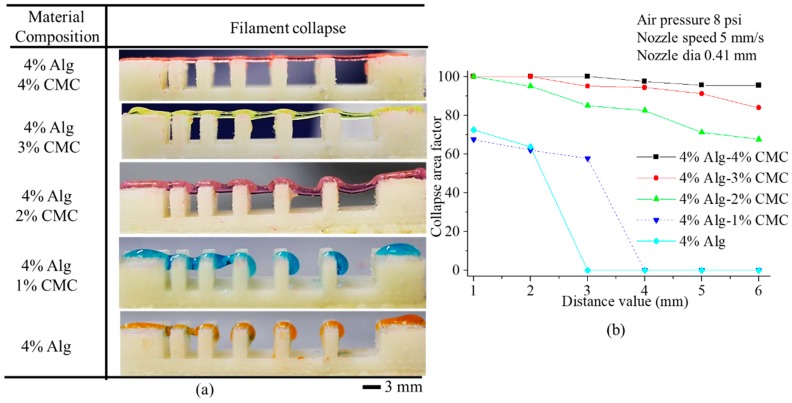
(**a**) Filament collapse test and (**b**) Collapse area factor.

The qualitative observation of the collapse test for various compositions of material identifies that with increasing the percentage of CMC leads minimum deflection of the suspended filament as shown in Figure 12a. Composition with 4% Alg-4% CMC generates almost straight filament on the pillars situated at various distances. The collapse area factor for each distance value of each material composition is determined using the Equation (4). With increasing the pillar to pillar distance, the collapse area factor shows decreasing trend as shown in Figure 12b. The graph indicates the composition with 4% Alg-4% CMC has minimum collapse area factor even with increasing the distance value. Composition with 4% Alg-1% CMC is not able to hold the filament geometry and breaks away even at 4 mm distance value. The same scenario happens for 4% at 3 mm distance value. Hence, this event results in zero collapse area factors for these two compositions.

### 3.5. Effect of Nozzle Speed, Air Pressure and Print Distance on Filament Width

**Figure 13:**
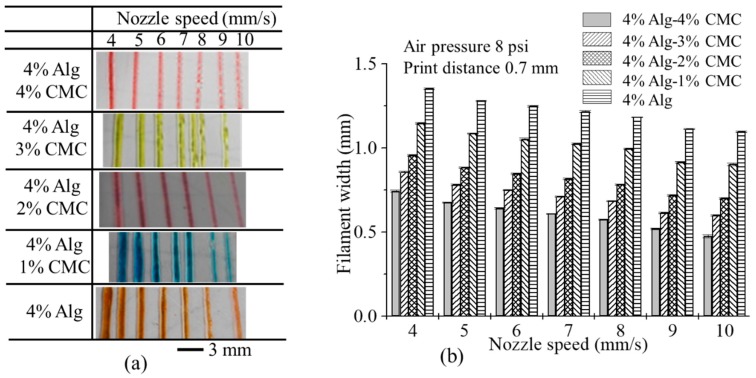
(**a**) Printed filaments at different nozzle speed; (**b**) the effect of nozzle speed on filament width.

The nozzle speed directly influences the filament width for all types of material compositions as shown in Figure 13a. With increasing the nozzle speed, the filament width reduces for all material compositions as shown in Figure 13b.

With increasing the nozzle speed, the suspended filament experiences tension along deposition direction and become thinner. Higher nozzle speed may break down the filament results in a discontinuous material deposition. For each composition, the filament width is greater than the nozzle diameter because of material expansion after extrusion. The addition of CMC with alginate reduces the expansion of material after extrusion which results in less difference of the filament width with respect to original nozzle diameter at various nozzle speeds as shown in Figure 13b. Hence, composition with 4% Alg-4% CMC shows minimum filament width than other compositions.

**Figure 14:**
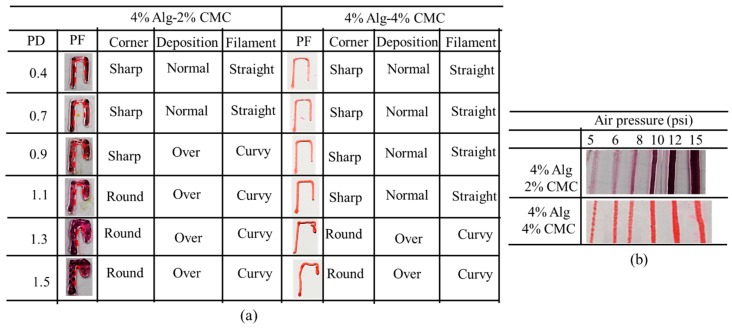
Qualitative effect of (**a**) print distance; (**b**) air pressure.

**Figure 15:**
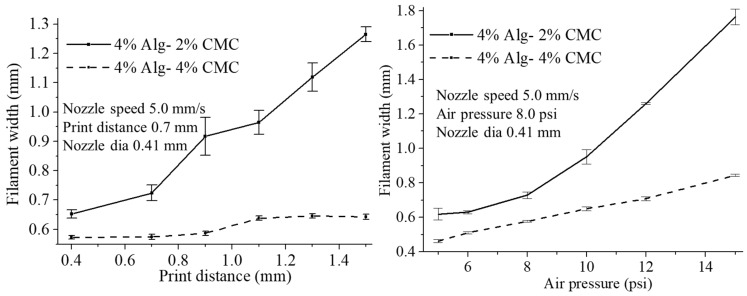
Effect of (**a**) Print distance; (**b**) Air pressure.

The effect of print distance on filament width is shown in Figure 14a and Figure 15a. With increasing the print distance, the filament width is increasing. Apparently, the corner of the deposited filament changes from sharp to round with increasing print distance. Filament deposited from a higher distance get less contact with the build plane which leads the starting point of the deposition further away and results in accumulation of material. This event also creates curvy filament as shown in Figure 14a. With increasing the portion of CMC, the changes in filament width reduces with increasing print distance. Composition with 4% Alg-4% CMC shows a better corner and filament geometry and material deposition than a composition with 4% Alg-2% CMC.

With increasing the air pressure, the filament width of 4% Alg-2% CMC and 4% Alg-4% CMC composition increases as shown in Figure 14b and Figure 15b. However, the increased amount of CMC into alginate will reduce the changes of the filament width as shown in Figure 15b. Applying less air pressure may not exceed the surface tension of the material with the nozzle and eventually will not deposit continuous filament. Besides, over material deposition may happen due to high air pressure. Thereby, applying optimum air pressure for different material compositions will assure the continuous filament deposition as well as uniform filament width.

### 3.6. Qualitative and Quantitative Test of Lateral Pore

**Figure 16:**
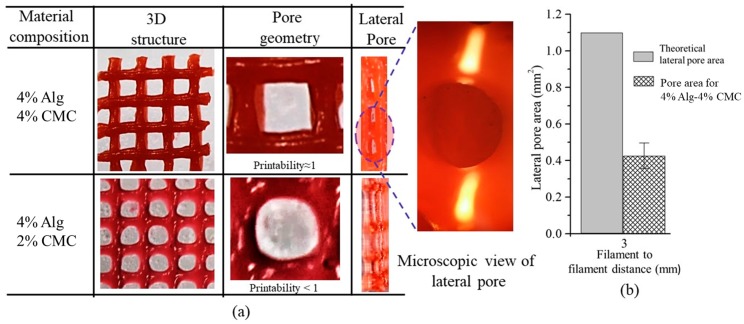
(**a**) Qualitative and (**b**) Quantitative test for lateral pore.

Scaffolds having a filament to filament distance of 3 mm are fabricated (10 layers) with compositions of 4% Alg-4% CMC and 4% Alg-2% CMC as shown in Figure 16a. Qualitatively, each pore of the scaffold fabricated by former composition is almost square. On the other hand, the pore geometry of scaffold fabricated by later composition is almost circular. To visualize the lateral pore in the scaffold structure clearly, the scaffold is cut laterally and observed the lateral pore macroscopically and microscopically. The presence of lateral pore in the scaffold fabricated by 4% Alg-4% CMC is quite clear. However, due to the higher collapse of the filament and comparatively less viscosity, lateral pore in the scaffold fabricated by 4% Alg-2% CMC was almost invisible. Therefore, it can be claimed that scaffold fabricated by 4% Alg-4% CMC maintains the lateral pore as well.

For given 3 mm filament to filament distance and 0.41 mm nozzle diameter, the theoretical lateral pore area is calculated as 1.1 mm^2^. The lateral pore area presented in the scaffold fabricated by 4% Alg-4% CMC is measured (*n* = 5) and compared with theoretical pore area. Using Equation (5), the lateral collapse area factor is determined as 37% for this specific filament to filament distance and nozzle diameter. Since, there was no lateral pore in the scaffold fabricated with 4% Alg-2% CMC, lateral collapse area factor for this composition is zero.

### 3.7. Scanning Electron Microscopy (SEM)

**Figure 17:**
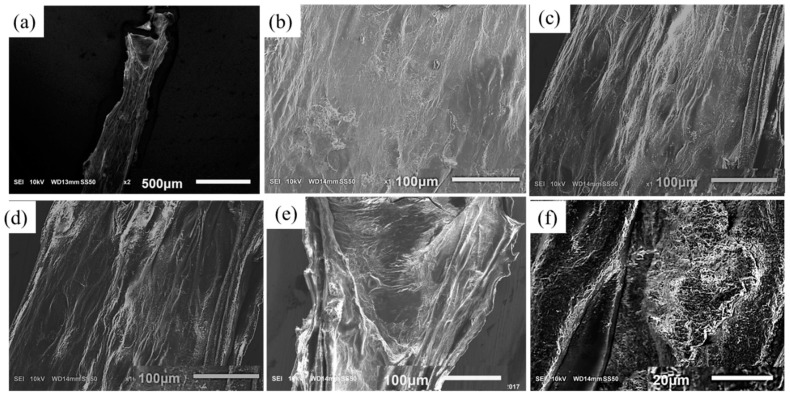
Scanning electron microscopy of filament with (**a**,**b**) 4% Alg and (**c**) 4% Alg-4% CMC before incubation; (**d**–**f**) 4% Alg-4% CMC after 3 days of incubation.

To study the influence of CMC on the filament microstructure, SEM imaging is performed with novel hybrid hydrogel 4% Alg-4% CMC in comparison to pure 4% alginate as shown in Figure 17. The cross-linked filament surface of pure alginate is smooth because of forming a primary membrane with Ca^2+^ as shown in Figure 17b. On the other hand, the filament surface fabricated with Alg-CMC hydrogel is highly porous as shown in Figure 17c assists potentially more to supply the nutrients and oxygen to the encapsulated cell. The filament incubated 3 days showing more pores as shown in Figure 17d–f indicating more release of CMC.

### 3.8. Swelling Test of Filament

**Figure 18:**
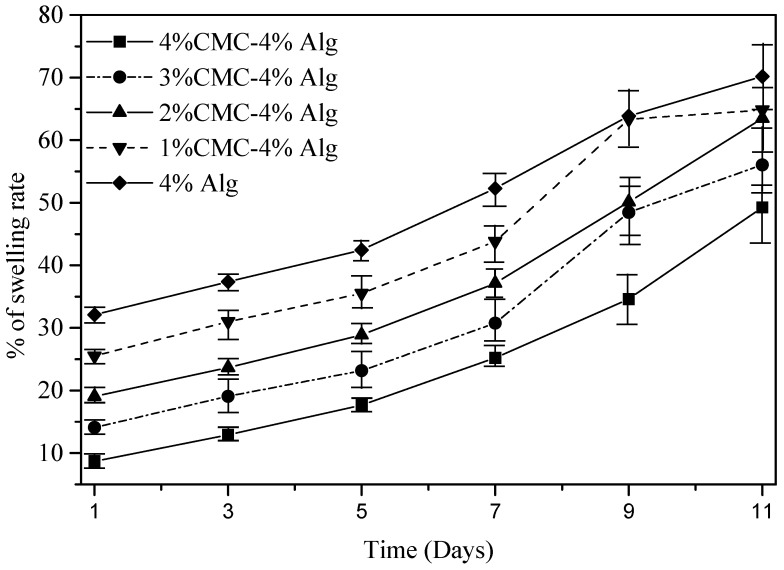
Swelling rate of different compositions of material.

All the compositions of material exhibit an upward trend of the swelling rate with the changes of time as shown in Figure 18. The mean swelling rate of each material combination at different time periods, that is, at day 1, 3, 5, 7, 9, 11 is significantly different (*p*-value = 0 with 95% confidence interval). Likewise, various material compositions show a significant difference in swelling rate among them as shown in Figure 18. Reducing the percentage of CMC increases the swelling rate and pure alginate shows maximum swelling. The reason behind this behavior is the addition of CMC increases the crosslinking density. Hence, the water absorption decreases and eventually the swelling rate.

### 3.9. Large-Scale Scaffold Fabrication

**Figure 19:**
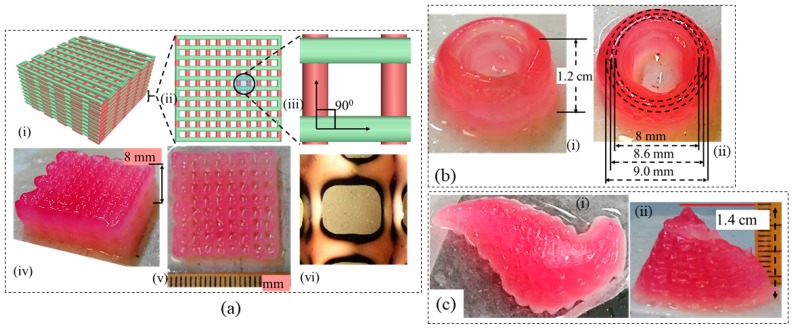
Large-scale scaffold fabricated by 4% Alg-4% CMC (**a**) CAD design (i–iii) with 0–90 deg pattern and corresponding fabrication (iv–vi); (**b**) a thin wall cylinder; (**c**) Pancreas structure from anatomical model.

Once the aforementioned characterization tests are done, it is revealed, the composition of 4% Alg-4% CMC shows good printability and shape fidelity. Therefore, this composition is used to fabricate acellular large-scale scaffolds as shown in Figure 19. The represented scaffold figures demonstrate the capability of this composition to hold the shape fidelity of large-scale scaffold fabrication.

### 3.10. Analysis of Cell Viability

**Figure 20:**
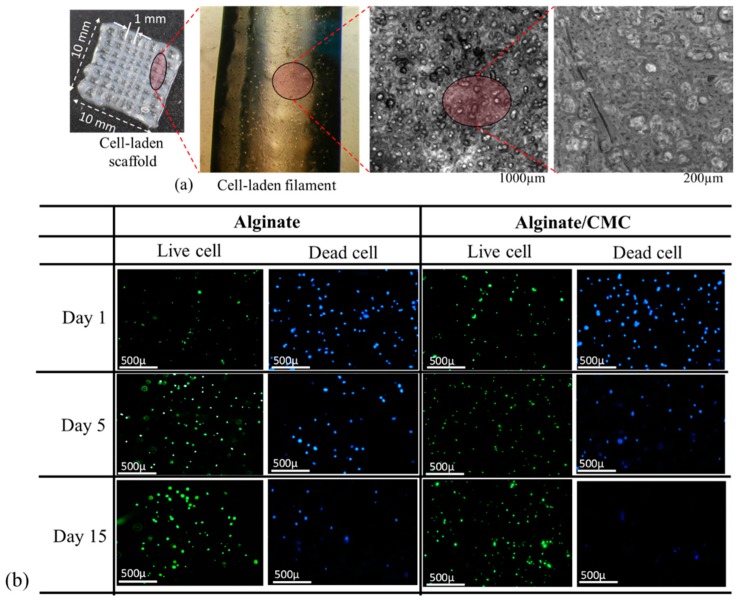
(**a**) Cell-laden scaffold and filament and (**b**) Live/dead staining of BxPC3 cell encapsulated in alginate and Alg-CMC hydrogel at different time periods.

**Figure 21:**
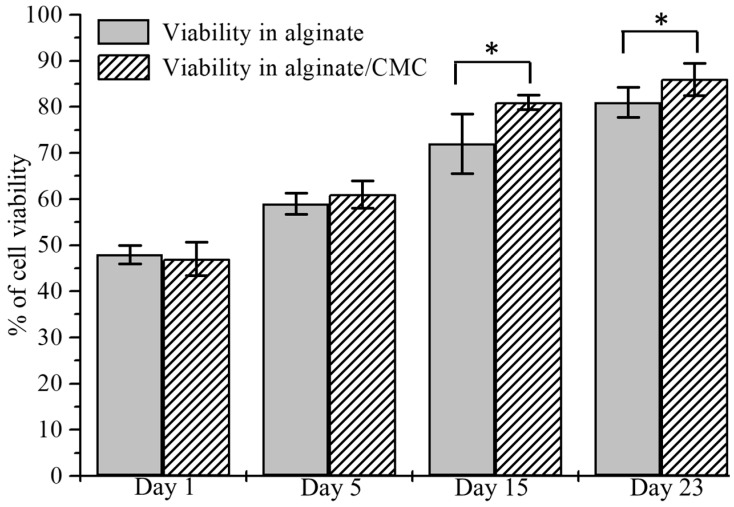
Comparison of cell viability into alginate and Alg-CMC at the different time.

BxPC3 cells are mixed uniformly with pure alginate and 4% Alg-4% CMC using magnetic stirrer immediately prior to dispensing the hydrogel to fabricate the scaffold. Viability at different predefined time periods, that is, day 1, 5, 15, 23 using staining of the live/dead cell is determined. A significant amount of live and dead cell is observed after dispensing and cross-linking the hydrogels with CaCl_2_ as shown in Figure 20b. At the first day, the percentage of viability for alginate and alginate/CMC was almost similar and there was no significant difference. However, the cell viability into an alginate-CMC blend was higher and statistically different than into alginate at day 15 and 23 as shown in Figure 21. From the SEM image, it is quite clear that presence of CMC induces more porosity into the structure over time. Thereby, the cell-laden filament fabricated with 4% Alg-4% CMC allows more exchange of required growth factor and removal of waste material which drives towards higher cell viability.

**Figure 22:**
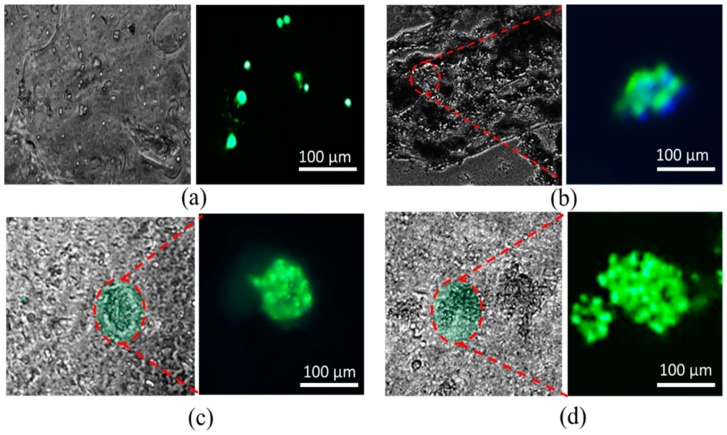
Generation of cell spheroid at day (**a**) 1; (**b**) 5; (**c**) 15 and (**d**) 23.

The morphology of BxPC3 cell is observed by phase contrast at different time periods into alginate and alginate/CMC. Minimum of five spots of spheroid morphology is visualized in each sample of alginate/CMC and the diameter of the spheroid is continued to increase as shown in Figure 22.

## 4. Conclusions

In this research, a novel hybrid hydrogel, that is, sodium alginate with carboxymethyl cellulose (CMC) is developed and optimized for 3D printing application. A set of systematic quantitative characterization tests are conducted to validate its printability, shape fidelity. The outcome of the rheological and mechanical test, filament collapse and fusion test demonstrate the favorable shape fidelity. Those characterization tests reveal that the composition of 4% Alg-4% CMC maintains good printability and shape fidelity for large-scale scaffold fabrication. Afterwards, 3D scaffold structures are fabricated with the pancreatic cancer cells, BxPC3 with these compositions and the cell viability is recorded 86% after 23 days which is comparable with pure alginate. To fabricate large-scale functional tissue scaffold, this hybrid hydrogel can be a potential biomaterial in 3D bioprinting process and the outlined characterization techniques open an avenue directing reproducible printability and shape fidelity. The future direction of this research is to fabricate large-scale cell-laden tissue scaffolds with different types of functional cell and determining the physiological behavior of co-culture into the microenvironment.
